# Validity of St Gallen risk categories in prognostication of breast cancer patients in Southern Sri Lanka

**DOI:** 10.1186/s12905-018-0524-1

**Published:** 2018-01-31

**Authors:** Harshini Peiris, Lakmini Mudduwa, Neil Thalagala, Kamani Jayatilake

**Affiliations:** 1 Medical Laboratory Science Degree Programme, Faculty of Medicine, University of Ruhuna, Galle, 80000 Sri Lanka; 2 Department of Pathology, Faculty of Medicine, University of Ruhuna, Galle, Sri Lanka; 3 Child Development & Special Needs Unit, National Child Health Programme, Family Health Bureau, Ministry of Health, No. 231, De Sarem Place, Colombo, 10 Sri Lanka; 4 Department of Biochemistry, Faculty of Medicine, University of Ruhuna, Galle, Sri Lanka

**Keywords:** Breast cancer, St Gallen risk categories, Survival, Sri Lanka

## Abstract

**Background:**

Although, there are many developments in the field of management, breast cancer is still the commonest cause of cancer related deaths in women in Sri Lanka. This emphasizes the need for validation of treatment protocols that are used in Sri Lanka for managing breast cancers. There are no published papers on treatment and survival of breast cancer patients in Sri Lanka. Hence this study was designed to determine the validity of St Gallen risk categories based on the survival outcomes of breast cancer patients in Southern Sri Lanka.

**Method:**

This retro-prospective study included all female breast cancer patients who had sought the immunohistochemistry services of our unit from May 2006 to December 2012. Patients who had neo-adjuvant chemotherapy were excluded. Patients were stratified according to the St Gallen risk categories; low-risk (LR), intermediate-risk (IR) and high-risk (HR), which is used in deciding on the adjuvant treatment. IR category was subdivided based on presence/absence of 1–3 positive-nodes (absent-IR1, present-IR2) and HR on the number of positive-nodes(1–3 lymph nodes;HR1,> 3 lymph nodes;HR2). Kaplan-Meier and Cox-regression models were used in the survival analysis.

**Results:**

This study included 713breast cancer patients (LR-2%, IR1–45%, IR2–10%, HR1–13%, HR2–30%). Five year breast cancer specific survival (BCSS)wasLR-100%, IR-91%, HR-66% and the recurrence free survival (RFS) was LR-85%, IR-84%, HR-65%. BCSS and RFS curves were significantly different between the three risk categories (*p* < 0.001).

No survival difference was evident between the IR1 and IR2 (BCSS-*p* = 0.232, RFS-*p* = 0.118). HR1 and HR2 had a distinctly different BCSS (*p* = 0.033) with no difference in RFS (*p* = 0.190).

**Conclusion:**

This study validates the St Gallen risk categorization of female breast cancer patients in our setting. However, the HR includes two subsets of patients with a distinct difference in BCSS.

## Background

The incidence of breast cancer has increased during the last decade to become the commonest cancer in Sri Lanka. Since the year 2000, breast cancer is the commonest cancer among females in Sri Lanka accounting for the highest cancer mortality [1–3]. The age standardized death rate for females with cancer was 53.5 per 100,000 population in 2010 [3].The majority of the breast cancer patients present with poor prognostic features [4]. Only a very few males are affected while 98.5% are females [3]. Five year overall survival and the breast cancer specific survival (BCSS) of the breast cancer patients in Southern Sri Lanka was 76 and 78% respectively [5]. To improve the survival of patients, adjuvant treatment is administered after primary surgical management. Adjuvant treatment includes local irradiation after mastectomy, systemic therapy with cytotoxic drugs, endocrine therapy and targeted therapy [6].

In Sri Lanka too, therapeutic decisions for breast cancer patients are based on the recommended guidelines that are being practiced worldwide. St Gallen International Expert Consensus on the primary therapy for early breast cancer is widely used by many oncologists [7–10].

St Gallen international consensus panel of experts developed a series of guidelines and recommendations for selection of adjuvant systemic treatment for breast cancer patients based on risk categories [7]. Initially patients were categorized into three risk groups; low risk, intermediate risk and high risk depending on the nodal status [7]. Later on more features have been added to this stratification and the modified version was published in 2007 [9].

The low risk category included patients who had node negative breast cancers with all good prognostic features; tumour ≤2 cm in size, grade 1, no lympho-vascular invasion, age ≥ 35 years and negative for human epidermal growth factor receptor 2 (HER2).The intermediate risk category consists of patients with either node negative and poor prognostic features (*p*T > 2 cm or grade 2–3 or presence of lympho-vascular invasion or HER2 positive or age < 35 years) or node positive (1–3 involved nodes) with good prognostic features (oestrogen receptor (ER) and/or progesterone receptor (PgR) positive and HER2 negative). Patients who had 1–3 positive nodes and HER2 positive or > 4 nodes positive breast cancers are categorized into high risk group.

Adjuvant systemic treatment regimens are decided based on these risk groups. Basically chemotherapy is recommended for patients who are at intermediate or high risk while only endocrine therapy is recommended for patients at low risk with expression of hormone receptors [7].

In addition to clinical risk categories, TNM (tumour-node-metastasis) stage is used to group patients to determine the treatment algorithm and prognosis. The TNM staging system identifies four degrees of tumour size, four degrees of lymph node status and two degrees of metastasis forming 19 TNM categories [11]. The combination of homogeneous groups with respect to survival makes four groups; stage I, II, III and IV [12].

Although, there are many developments in the field of management, breast cancer is still the commonest cause of cancer related deaths in women in Sri Lanka [3].This emphasizes the need for validation of treatment protocols that are used in Sri Lanka for managing breast cancers. There are no published papers on retrospective studies or clinical trials on treatment and survival of breast cancer patients in Sri Lanka. Hence, this study was designed to determine the validity of St Gallen risk categories based on the survival outcomes of breast cancer patients in Southern Sri Lanka.

## Method

This was a retrospective study with a prospective patient follow-up. Of the 1068female breast cancer patients who had sought the Immunohistochemistry (IHC) services of the Diagnostic Immunohistochemistry Laboratory of the Department of Pathology of our institution from May 2006 to December 2012, only 944 patients gave consent to participate in the study. This unit was the only immunohistochemistry laboratory that was available to cater to the cancer patients of Southern Sri Lanka from 2006 till the completion of the study. Therefore, the study sample represented the female breast cancer patients in the geographic area mentioned. This study was approved by the Ethical Review Committee of our institution.

The histopathological features of breast cancers were retrieved from the laboratory records available in the laboratory. Nottingham grading of all breast cancers were done by a single investigator using the haematoxylin and eosin (H&E) stained slides to eliminate inter-observer variation. Nottingham Prognostic Index (NPI) was calculated for all breast cancers using the formula; NPI = 0.2 × tumour size (cm) + lymph node stage (1, 2 or 3) + histological grade (1, 2 or 3) [13].

### Laboratory methods

The ER, PgR and HER2 expressions were evaluated using the immunohistochemically stained slides retrieved from the archives of the department. For IHC staining of all breast cancers to assess the ER, PgR and HER2 expression, primary monoclonal mouse antihuman estrogen receptor α clone 1D5 (Dako-M7047), monoclonal mouse antihuman progesterone receptor (Dako- M3569) and polyclonal rabbit antihuman c-erbB-2 oncoprotein (Dako-A0485) respectively have been used with the secondary antibody (Dako Real EnVision™). The Allred score was used to assess ER and PgR status. The UK recommendations were used for the assessment of HER2 expression [14]. IHC assessment was done by a single investigator in order to eliminate inter-observer bias. Patients who had no staining for ER, PgR and a score of 0 or + 1 for HER2 were considered as triple negative (TNBC). Breast cancers were scored as ER/PgR positive if the total Allred score for ER/PgR was > 2/8. A score of + 3 was considered positive for HER2 over expression. Patients who had + 2 for HER2 and found to be positive by FISH were also included as HER2 positive.

### Follow up and outcomes

After enrolling, the study subjects were followed up for recurrence or death at 6 month intervals. The study ended on 31st December 2013. The mean follow-up time was 47 ± 23 months. The actual minimum follow up period was 12 months. One third of the total population was followed up beyond four years from the date of diagnosis (83% for 24 months, 60% for 36 months, 44% for 48 months and 33% for five or more years).

The BCSS time was calculated from the date of diagnosis of the disease to the date of death from breast cancer or death with breast cancer [15]. Deaths from other causes or from unknown causes were censored to the date of death. The cause of death of the patient was obtained from the death certificate issued by the Department of Registrar General, Sri Lanka.

Recurrence free survival (RFS) time was calculated from the date of surgery to the date of diagnosis of breast cancer recurrence which included loco-regional and distant recurrences [15]. Radiological and histopathological data were used to confirm the recurrence. The date of the said investigation was considered the date of recurrence. Second primary cancer and in situ carcinomas were not included as an event. Patients who died before a recurrence were censored to the date of death [15].

### Statistical analysis

All the study subjects were categorized according to the St Gallen risk stratification; low risk, intermediate risk and high risk [9] except the patients who had neo-adjuvant chemotherapy.

The intermediate risk and high risk categories were subdivided. The intermediate risk subgroup-1 included node negative breast cancers with at least one of the six poor prognostic features; tumour size > 2 cm, grade 2 or 3, presence of lympho-vascular invasion, no ER/PgR expression, HER2 over expression and age < 35 years. Intermediate risk subgroup-2 included patients who had 1–3 positive lymph nodes with better prognostic features; tumours that were both ER and PgR positive and negative for HER2. The high risk subgroup-1 included patients with 1–3 positive lymph nodes with either absence of ER and PgR expression or HER2 positivity. All breast cancers with four or more positive lymph nodes were categorized into high risk subgroup-2 irrespective of the other prognostic features.

Kaplan-Meier model was used to estimate the 5 year BCSS and RFS of patients who were at low risk, intermediate risk, high risk and in sub groups of intermediate risk and subgroups of high risk. Difference in the survival was compared with the log-rank test. A *p* value < 0.05 was considered statistically significant.

## Results

A total of 713 patients were included in this study. Most of the patients had undergone mastectomy as the surgical mode of management (99%).The clinico-pathological features of the study cohort at presentation are tabulated in Table 1. The majority of the patients had poor prognostic features at presentation. Therefore majority of them were either in intermediate risk category (396, 55%) or high risk category (302, 43%). Only 15 patients (2%) were in the low risk category.

**Table 1:** Clinico-pathological features of the study cohort

Clinico-pathological features	*n*(%)	Clinico-pathological features	*n*(%)
Tumour size		TNM stage	
< 20 mm	219 (30.7)	Stage I	133(18.6)
> 20-50 mm	425 (59.6)	Stage II	341(47.8)
> 50 mm	55 (7.7)	Stage III	225(31.5)
Unknown	14 (1.9)	Stage IV	5(0.7)
Nottingham grade		Unknown	09(1.2)
Grade 1	71(10)		
Grade 2	279(39.1)	Expression of ER	
Grade 3	291(40.8)	Positive	241(33.8)
Unknown	72^a^(10)	Negative	409(57.4)
Lymph node stage		Unknown	63^a^(8.8)
Stage 0	336(47.1)		
Stage 1	163(22.8)	Expression of PgR	
Stage 2	134(18.8)	Positive	259(36.3)
Stage 3	80(11.2)	Negative	386(54.1)
		Unknown	68^a^(9.5)
Lympho-vascular invasion			
Presence	218(30.5)	Expression of HER2	
Absence	494(69.5)	Positive	133(18.6)
Unknown	01(0.1)	Negative	484(67.9)
		Unknown	96^a^(13.5)

*n* number, % percentage, *ER* oestrogen receptor, *PgR* progesterone receptor, *HER2* human epidermal growth factor receptor2

^a^ Grade, ER, PgR and HER2 could not be assessed as the archival slides and tissue were poorly preserved

Majority of the intermediate risk and high risk patients had received a combination of chemotherapy, radiotherapy, endocrine therapy and targeted therapy. Only 14 patients (*n* = 14) had refused to take any adjuvant treatment; chemotherapy, radiotherapy or targeted therapy.

Out of all patients in intermediate or high risk category, 91% of patients had received the complete course of adjuvant chemotherapy. There were three main poly-chemotherapy regimens used for the present cohort; AC (doxorubicin, cyclophosphamide), CMF (cyclophosphamide, methotrexate and 5-fluorouracil) and FEC (5-fluorouracil, epirubicin and cyclophosphamide). In addition, taxol had been recommended alone or in combination with the above poly-chemotherapy regimens.

Patients who had expression of ER and PgR had received either tamoxifen (95%) or aromatase inhibitors (2%) or both (3%) as hormone therapy. Radiotherapy had been given to 71.4% of the patients in the present study. Forty four percent of the patients who had HER2 positive tumours had received antiHER2 therapy. In 2006, 10% of patients who had Her2 positive breast cancers had received anti Her2 therapy and it had risen to 78% in 2012.

**Fig. 1:**
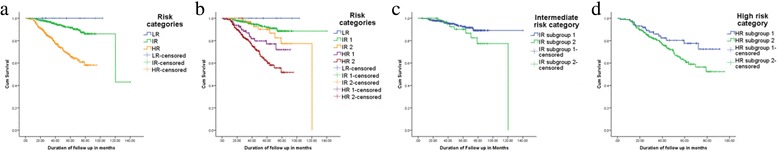
Breast cancer specific survival by; **a** the three risk categories; **b** the three risk categories with the subgroups; **c** thesubgroups of the intermediate risk category and **d** the subgroups of the high risk category. Total = 713, low risk (LR) = 15, intermediate risk(IR) = 396 (subgroup 1 = 321, 2 = 75), high risk subgroup (HR) = 302 (subgroup 1 = 88, 2 = 214); total events = 106; log-rank **a** *p* < 0.001; **b** *p* < 0.001; **c** *p* = 0.232; **d** *p* = 0.033

**Fig. 2:**
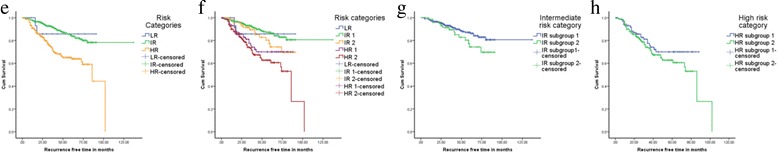
Recurrence free specific survival by; **e** the three risk categories; **f** the three risk categories with the sub groups; **g** the subgroups of the intermediate risk category and **h** the subgroups of the high risk category. Total = 705, low risk (LR) = 15, intermediate risk(IR) = 392 (subgroup 1 = 317, 2 = 75), high risk subgroup (HR) = 298 (subgroup 1 = 87, 2 = 211); total events (recurrences) = 136. Log-rank **e*** p* < 0.001; **f*** p* < 0.001; **g*** p* = 0.118; **h*** p* = 0.190

The patients who were in the low risk category had the best expected 5 year BCSS (100%) while who were in the high risk category had the lowest 5 year BCSS (66%). Five year RFS of the patients who were in the low risk, intermediate risk and the high risk categories were 85, 84 and 65% respectively. (Figs. 1 and 2).

The BCSS and RFS curves were significantly different between the three risk categories (*p* < 0.001) and three risk categories with their subgroups too. No survival difference was evident between the intermediate risk sub group 1 and 2 (BCSS *p* = 0.232; RFS *p* = 0.118). The high risk sub group 1 and 2 had a significantly different BCSS (*p* = 0.033). However, RFS was not significantly different (*p* = 0.119) between the high risk subgroup 1 and 2. (Figs. 1 and 2).

**Fig. 3:**
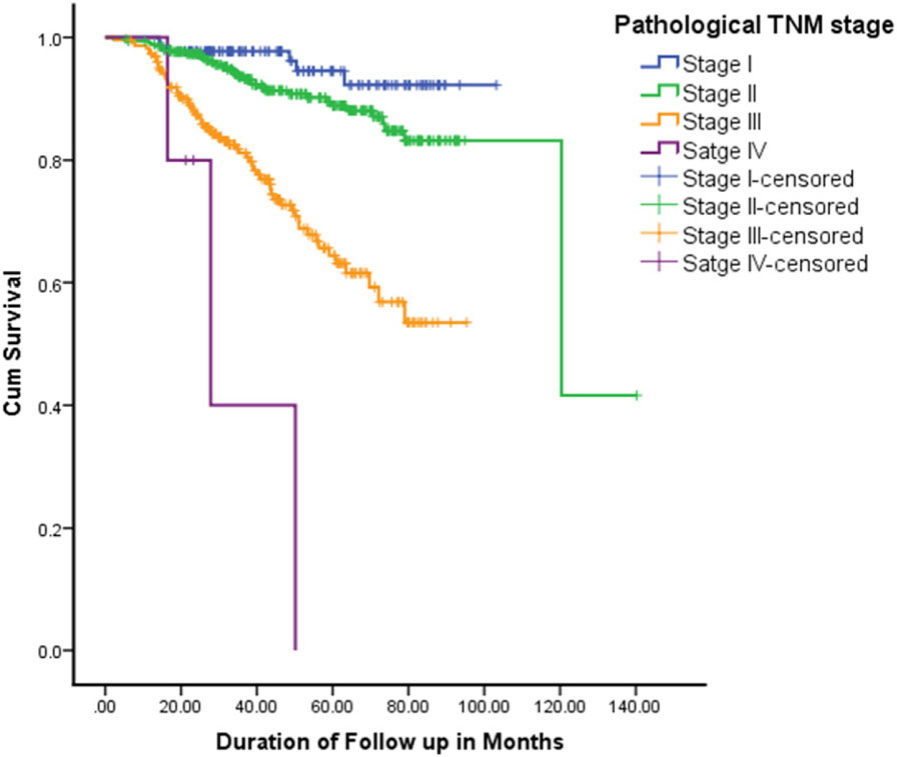
Breast cancer specific survival of the patients by TNM stage. Total = 704; stage *I* = 133, II = 341, III = 225 and IV = 5; total events = 105. Log rank test *p* < 0.001

**Fig. 4:**
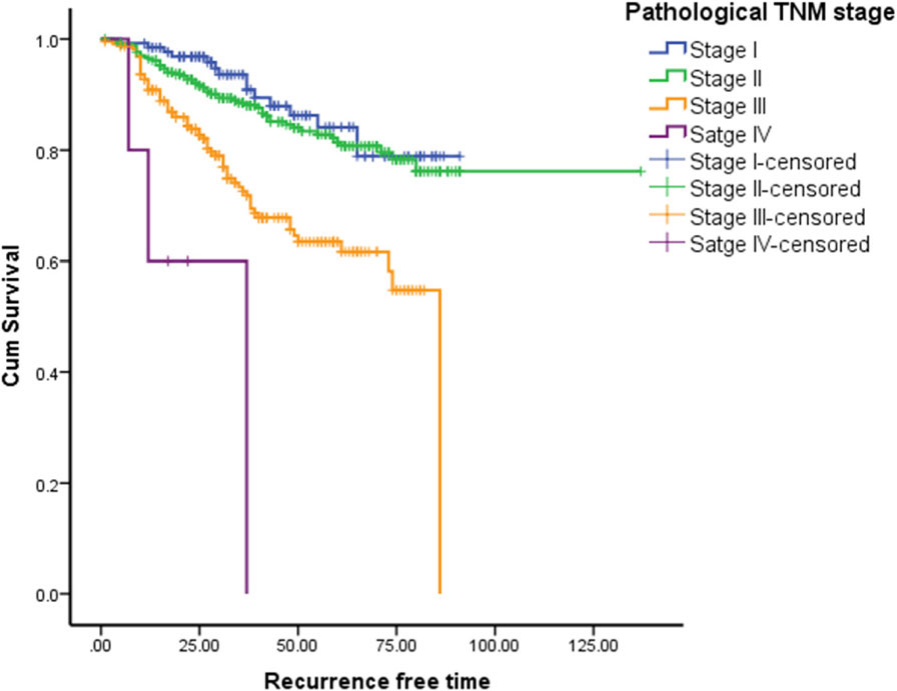
Recurrence free survival of the patients by TNM stage. Total = 696; stage *I* = 130, II = 339, III = 222 and IV = 5; total events = 135. Log rank test *p* < 0.001

The BCSS and RFS of the cohort were estimated according to the TNM stage (Figs. 3 and 4). The 5 year BCSS of the patients who were in the stage I, II, III and IV were 94.5, 88.9, 64.4 and 0% respectively (*p* < 0.001). The 5 year RFS of the patients in stage I to IV were 84.2, 81.5, 63 and 0% respectively (*p* < 0.001).

The stage I patients were in the low risk group (*n* = 15) or in the intermediate risk group (*n* = 118). Stage II patients were in the intermediate risk group (*n* = 262) or in the high risk group (*n* = 79). Stage III patients were in the intermediate risk group (*n* = 8) or in the high risk group (*n* = 217). All the stage IV patients were in the high risk group. The stage II to IV included either intermediate or high risk patients.

## Discussion

In Sri Lanka, breast cancer is the commonest cancer among females since the year 2000. Most of the breast cancers have found to be of high grade with the absence of hormone receptor expressions [4, 16, 17]. The prevalence of hormone receptor positive breast cancers is on par with the previous studies done in Sri Lanka [16, 17] and it is less compared to the world figures [18]. Therefore, advance therapeutic options had been decided for the management of breast cancers with poor prognostic features.

The management of breast cancer is aimed to achieve good local control of both the primary tumour and the regional nodes in the axilla and to reduce the subsequent risk of relapse and death. In the present study, 99% of the patients had undergone mastectomy with axillary clearance up to the level II as the surgical management although the trend in western world and in some specialized centers in Sri Lanka is to have limited surgery conserving the figure of females. They had received systemic therapy and/or radiation as adjuvant treatment. Adjuvant systemic treatment regimens for the breast cancer patients in this study cohort had been decided mostly based on the St Gallen risk categorization.

For making adjuvant therapeutic decisions for early stage breast cancer, different types of clinical guidelines are used. Current clinical guidelines recommend tumour biomarkers (ER, PgR, HER2, Onco*type* DX, Endo Predict, PAM50), pathological features (eg: TNM staging system) and risk stratification tools. The American Society of Clinical Oncology (ASCO) and Cancer Care Ontario (CCO) guidelines recommend Onco*type* DX and Adjuvant! Online to make adjuvant therapy decisions [19]. The above techniques which are included in the guidelines are not practiced in our setting, routinely. A simple low cost tool is the best option for the management of breast cancer patients in Sri Lanka as it is a low-middle income country. A set of guidelines and recommendations for selection of adjuvant systemic treatment based on risk categories developed by the experts in the St Gallen international consensus panel can be easily used in our setting [7, 9]. Further, it provides clinically useful breast cancer treatment consensus for the patients treated outside the clinical trials in most countries [20].Even though St Gallen risk stratification is used for the management of breast cancer, it has not been validated in our setting. Therefore, this study was designed to eliminate this lapse.

In the present study, only 2% were at low risk and they had a 100% 5 year BCSS. The total number of patients included in the low risk category was small and therefore the 5 year BCSS obtained may be an over estimation. The low risk category included patients who had node negative breast cancers with all good prognostic features; tumour ≤2 cm in size, grade 1, no lympho-vascular invasion, age ≥ 35 years and negative for HER2 [9]. Previous studies too, have found that node negative breast cancers have good prognosis compared to the node positives irrespective of the other factors [21, 22].

In the current study cohort, majority of the patients had poor prognostic features. Therefore, they belonged to intermediate or high risk categories. In the current study, the two subgroups of the intermediate risk category did not have a significant survival difference (Figs. 1 and 2). The combinations of adverse and good prognostic features of the sub groups of intermediate risk category seem to have nullified the effect of each other giving a similar prognosis to both subgroups and hence similar survival curves. This study justifies the selection criteria for intermediate risk category.

Patients who had 1–3 positive nodes and HER2 expression or > 4 nodes positive breast cancers are categorized into high risk group. However, the high risk category which has the worst survival included two subsets of patients with a distinct difference in BCSS. This difference was imparted by the selection criteria for the high risk category. The high risk category sub group 1 included patients with 1–3 positive lymph nodes and at least one of the poor prognostic features while high risk category subgroup 2 included patients with four or more positive lymph nodes irrespective of the other prognostic features. Therefore, this study emphasizes the worst survival of patients with four or more positive lymph nodes irrespective of any other factor.

Similarly, a study done in USA based on St Gallen risk categories has stated that there was no significant difference between the 5 year relative survival of two intermediate risk categories. Those with four or more positive lymph nodes (high risk sub group 2) had poor survival compared to the high risk sub group 1 [23].

A significant RFS difference was observed only among the three main risk categories. The RFS of the subgroups of intermediate risk or high risk categories did not show a significant difference. The factors which were considered for the sub grouping of intermediate and high risk categories have not significantly affected the RFS of the particular sub group. Therefore, intermediate and high risk categories are homogenous with regard to the RFS.

The majority of the female patients in the current study had stage II or above breast cancers at presentation. The BCSS of the stage I which includes good prognostic features had the highest BCSS and RFS compared to the other stages. However, the BCSS/RFS of the patients in stage I-IV had lower 5 year survival rates compared to the clinical risk categories, indicating overlap in survival outcome among the four stages. Therefore this study suggests the risk categorization of patients over TNM staging as the risk category better identifies the outcome survival.

The study subjects were enrolled retrospectively for the current study. Therefore, it carries inherent limitations of retrospective studies. Use of past records of the patients and archived tissue blocks for re-assessment are associated with data loss. However, we included a substantially large number of sample at the study designing level expecting some amount of missing data. Further, the cohort of female breast cancer patients described in this manuscript has been treated at a single oncology unit in the Southern Sri Lanka. Therefore, the management of the study subjects can be considered uniform even though it may have differences compared to other institutions in Sri Lanka. In 2006, a lower percentage of HER2 positive breast cancer patients had received trastuzumab compared to the number of patients who received trastuzumab in 2012, towards the end of the study period. This was due to the limited availability of the drug in the public health sector during the said period. The poor survival of HER2 positive breast cancers patients who did not have trastuzumab may have affected the survival rates of the cohort.

## Conclusion

The current study validates the use of three main risk categories in our setting, as they have distinct BCSS and RFS. The intermediate risk group is a homogenous group irrespective of the inclusion of node-positive patients. The high risk category includes two subsets of patients with a distinct difference in BCSS substantiating heterogeneity of the high risk category. Therefore four risk categories; low risk, intermediate risk and high risk subgroup 1 and 2 with distinct survival difference were identified within the cohort of patients.

This study recommends the use of St Gallen risk categorization for the management of breast cancer patients in Sri Lanka and highlights the need to investigate further on the two subsets of patients in the high risk category.

## Abbreviations

BCSS: Breast cancer specific survival
ER: Oestrogen receptor
H&E: Haematoxylene & Eosin
HER2: Human epidermal growth factor receptor 2
NPI: Nottingham prognostic index
PgR: Progesterone receptor
RFS: Recurrence free survival
THC: Immunohistochemistry
TNBC: Triple negative breast cancer
TNM: Tumour-node-metastasis
